# Gastrointestinal adverse events of immunotherapy

**DOI:** 10.1259/bjro.20210027

**Published:** 2021-10-20

**Authors:** Giovanni Cappello, Francesca Molea, Delia Campanella, Francesca Galioto, Filippo Russo, Daniele Regge

**Affiliations:** 1 Candiolo Cancer Institute, FPO-IRCCS, Candiolo, TO, Italy

## Abstract

Cancer immunotherapy with immune-checkpoint inhibitors (ICIs) has emerged as an effective treatment for different types of cancer. ICIs are monoclonal antibodies that inhibit the signaling pathway that suppress antitumor T-cell activity. Patients benefit from increased overall and progression-free survival, but the enhancement of normal immunity can result in autoimmune manifestations, called immune-related adverse events (IRAEs), which may lead to a discontinuation of cancer therapy and to severe also life-threating events. IRAEs may affect any organs or system in the human body, being the gastrointestinal (GI) tract one of the most involved districts. Imaging plays an important role in recognizing GI IRAEs and radiologist should be familiar with the main spectrum of radiological appearance. Indeed, early detection of GI IRAEs is crucial for proper patient management and reduces morbidity and mortality. The purpose of this review is to present the most relevant imaging manifestation of GI IRAEs.

## Introduction

Immune modulators are an important class of relatively new anticancer drugs. By resetting the checks and balances that regulate T cell toxicity against tumors, these drugs increase patient’s overall and progression-free survival in several different clinical settings.^1,2^ William B. Coley, an orthopedic surgeon of the late 19th century, considered the father of immunotherapy (IT), was the first to envisage the role of the immune system in treating cancer, injecting a mixtures of bacteria (known as “Coley’s toxin”) in patients affected by bone sarcomas, inducing an immune antitumor response.^3,4^ Currently, we recognize two different types of IT: non-specific IT such as immune-checkpoint inhibitors (ICIs), which lead a general activation of the immune system without a specific antigen, and tumor-specific IT, such as oncolytic virus, cancer vaccines, and adoptive cell transfer, based on the response against a specific antigen.^5^ The first ICIs was approved by the U.S. Food and Drug Administration (FDA) in 2011 for the treatment of advanced melanoma.^6^ Since then, there has been a rapid expansion of the use of ICIs. Nowadays, immunotherapy is being used in the treatment of various types of tumors bringing about a paradigm shift in the management of oncologic patients.

### ICIs: mechanism of action

ICIs consist in a growing number of monoclonal antibodies (MAs) that target checkpoint molecules in T-cells or their ligands in antigen-presenting cells (APCs), tumor cells, and other cell types, which usually regulate the T-cell response to the antigen-major histocompatibility complex (MHC) on APCs.^7^ ICIs unbalance T-cell regulation by blocking the checkpoint molecules that normally inhibit T-cell activity directed against tumor cells or by activating in an agonist way the stimulation of one of the molecules that usually speed up T-cell-mediated tumor cell surveillance and destruction.^7^

**Figure 1.**
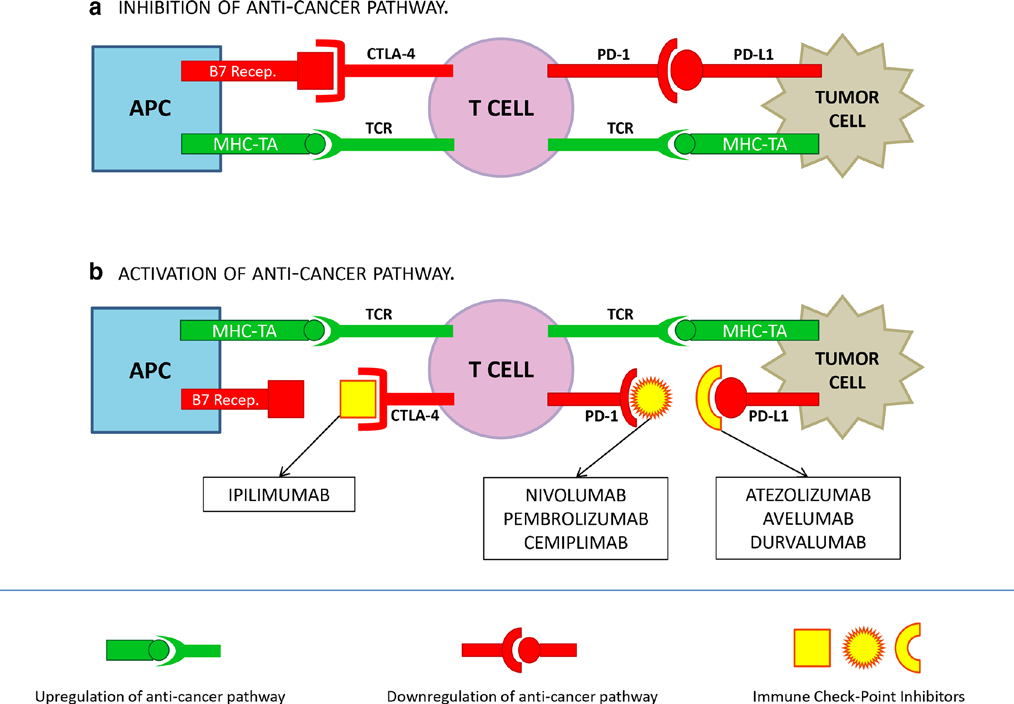
Mechanism of action of ICIs. The binding of PD-1/PD-L1 and CTLA-4/B7 receptor inhibit T cells response; the binding of MHC-TA and TCR activate T cell response. A: cancer cells reduce T cells response by up-regulating inhibition signals throughout the binding PD-1/PD-L1 and CTLA-4/B7 receptor. B: vice versa, the binding of ICIs to CTLA-4, PD-1 or PD-L1 prevent the activation of the anticancer pathway, by stimulating T cell response throughout MHC-TA/TCR binding. APC: Antigen Presenting Cell; MHC: Major Histocompatibility Complex; TA: Tumor Antigen; TCR: T Cell Receptor.

Seven ICIs are currently approved by the FDA for cancer treatment.^8^ The mechanisms of action are summarized in Figure 1. In brief, the most effective ICIs currently used in oncological clinical practice target three different molecules: cytotoxic T-lymphocyte associated antigen 4 (CTLA-4. FDA approved MA: Ipilimumab), programmed cell death protein-1 (PD-1. FDA approved MAs: Nivolumab, Pembrolizumab, Cemiplimab), and programmed cell death protein ligand-1 (PD-L1. FDA approved MAs: Atezolizumab, Avelumab, Durvalumab). CTLA-4 is a cell membrane protein, expressed on the surface of regulatory T cells, that interacts with B7 receptors expressed on the surface of APCs leading to an ”off switch” during the primary phase of T-cell activation. PD1 is a transmembrane glycoprotein expressed on the surface of immune-cells that binds PDL-1 expressed on the surface of tumor cells; the interaction between PD1 and PD-L1 down regulates the cytotoxic response of T cells.^9^ ICIs block CTLA-4/B7 receptor and PD-1/PDI-L1 pathways, enhancing T-cell action against tumor cells.

#### ICIs: immune-related adverse events (IRAEs)

By unbalancing the immune system, ICIs unselectively break self-tolerance to healthy tissues, favoring the development of autoimmune manifestations, called IRAEs.^9,10^ Pathophysiological mechanisms underlying IRAEs are still unknown, but are probably related to the loss of immunologic homeostasis with the potentiation of the effect of pre-existing autoantibodies.^11,12^ IRAEs are common events: in a review and meta-analysis by Wang et al, 66% of 18.610 patients from 106 studies developed at least one IRAE of any severity.^13^ The majority of IRAEs toxicity is manageable, but severe and life-threatening events may occur, leading to a discontinuation of therapy or to the hospitalization of the patients.^9^

The common terminology criteria for adverse events (CTCAE) is a descriptive terminology accepted throughout the oncology research community, born to standardize and grade IRAEs.^14^ The last version of CTCAE (version 5)^15^ divides IRAEs in five categories of increasing severity based on clinical manifestation, from Grade 1 to Grade 5, the latter being severe, corresponding to patient’s death.^15^ Besides the skin, the digestive tract is the most affected; however, IRAEs may involve almost any organ and system.^10^ The incidence of fatal events is estimated to be 0.3–1.3%.^16^ Radiologists must identify the most important imaging findings of gastrointestinal (GI) IRAEs in a timely fashion. Indeed, early detection and an immediate management of GI IRAEs usually avoid major morbidity and mortality. Moreover, recognizing IRAEs will avoid misinterpreting imaging findings for disease progression.^17^

### IRAEs – GASTROINTESTINAL TRACT

#### Incidence, clinical presentation, and management

**Figure 2.**
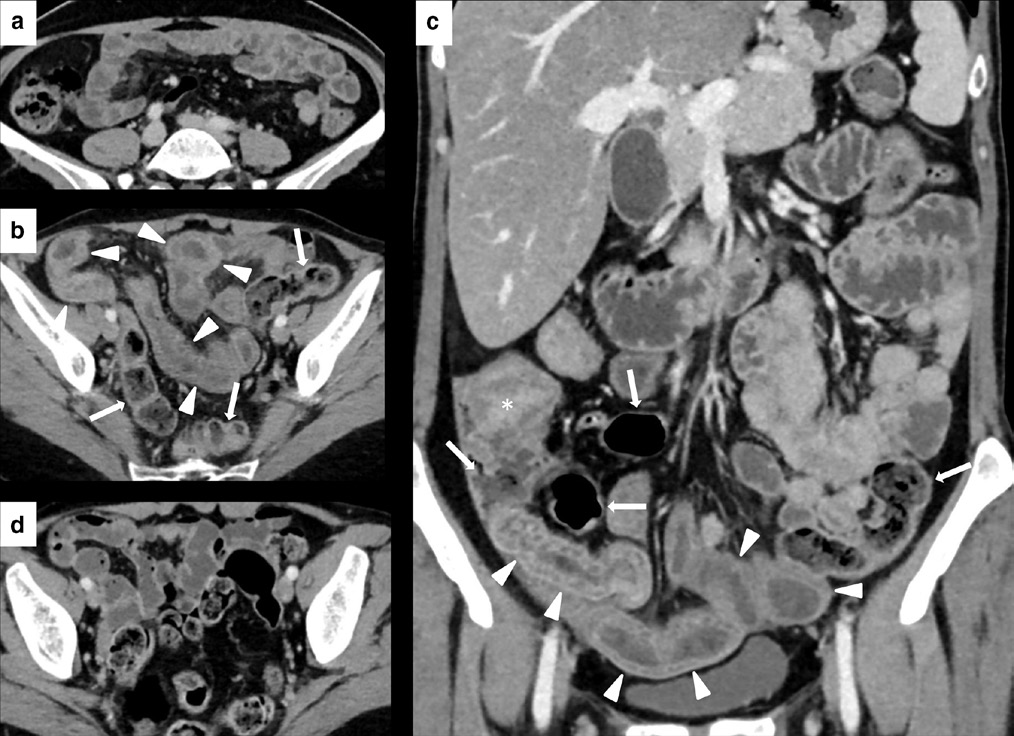
Imaging of ileitis without colitis in a 51-year-old female with metastatic breast cancer treated with Atezolizumab. Contrast-enhanced CT (CECT) at baseline did not show abnormalities of the last ileal loops wall (**A**). After 3 month of therapy, CECT showed a marked wall thickening, edema, and stratification involving the distal ileal loops (white arrowheads in B and C), without signs of colitis (white arrows in B and C, which show a regular colonic wall). After treatment discontinuation, CECT showed a remission of the ileitis (**D**). White asterisk in C shows the ileocecal valve.

The GI tract is the most commonly involved in case of Grade 3–5 IRAEs,^10^ the most important being enterocolitis, which frequently leads to treatment discontinuation. The most frequent manifestations of enterocolitis are colitis and diarrhea. The former includes symptoms such as abdominal pain, nausea/vomiting, cramping, blood or mucus in stool, while diarrhea is defined as increase of a stool frequency from baseline. Other symptoms are fever, abdominal distention, constipation, and weight loss. Life-threatening complications, such as bowel perforation, are present in severe cases.^16,18–20^ Diarrhea and colitis typically occur within 6–8 weeks from the beginning of treatment^21,22^ ; however, enterocolitis may manifest itself even several months after the end of therapy.^19^ The incidence is higher in case of anti-CTLA4 blockade, with diarrhea and colitis reported in 30–40%^23,24^ and 8–22% of cases,^19,24^ respectively. Fewer data are available on GI IRAEs incidence associated with anti-PD1/PD-L1 blockade, which are less frequent, while a higher incidence is described when different ICIs are combined in treatment.^19^ In a systematic review and meta-analysis, Wang et al reported an incidence of severe colitis and diarrhea of, respectively, 9.4 and 9.2% in patients receiving a combination of anti-CTLA-4 and anti PD-1 antibodies, 6.8 and 7.9% in patients treated with anti-CTLA4 antibodies monotherapy and only 0.9 and 1.2% in patients receiving PD-1/PD-L1 inhibitor monotherapy.^25^ According to the last version of CTCAE, the main parameter to stratify the severity of diarrhea and colitis is, respectively, the number of stools per day (or the ostomy output) and patient’s symptoms, both increasing proportionally with disease severity.^15^ The workup and management of the patients depend on the above reported severity scale and are summarized in Table 1. Although the colon is the most frequent site of IRAEs, ICI-related inflammation may also occur in the upper GI tract.^26^ Symptoms as nausea, vomiting, and abdominal pain usually coexist with lower GI tract symptoms and are widely non-specific. Indeed, they can be correlated also to concurrent cancer therapies or cumulative effect of previous treatment lines.^27^ Upper GI tract involvement has been reported in the form of esophagitis,^28–30^ gastritis,^28–34^ and duodenitis,^30,35^ often as case reports. ICI-induced ileitis without colitis is also an uncommon event^36–39^ (Figure 2). Management and treatment of upper GI IRAEs are similar to lower GI IRAEs.^27^

**Table 1.** Severity scale, management, and workup of lower GI IRAEs (^20,23^)

	CTCAE DEFINITION^16^		Work-up	MANAGEMENT		Therapy
	Severity of diarrhea	Symptoms of colitis		Anti PD-1/PD-L1	Anti CTLA-4 or combined therapy	
Grade 1	increase <4 stool per day or mild increase in ostomy output compared to baseline	asymptomatic	- Close monitoring	Continue ICI therapy Consider holding immunotherapy until symptoms subside		Hydration Antidiarrheal agents
Grade 2	increase of 4–6 stools per day or moderate increase in ostomy output compared to baseline	mild abdominal pain with mucus or blood in stool	Blood and stool work-up Tests for lactoferrin and calprotectin Screening laboratories^a^ Abdominal and pelvic Imaging (CT) GI endoscopy with biopsy	- Drug-free period until symptoms recover to G1	- Discontinue Anti CTLA-4 until symptoms recover to G1 and consider switch to anti-PD1/PD-L1 after resolution of toxicity	- Corticosteroids; if no response in 2–3 days consider increase dose or adding Infliximab
Grade 3	increase of ≥7 stools per day or severe increase in ostomy output compared to baseline	severe abdominal pain with/without peritoneal signs	Consider hospitalization As Grade 2 and consider repeating endoscopy for not responding patients			- Corticosteroids; if no response add infliximab; if no response consider vedolizumab
Grade 4	life threatening consequences			- Permanently discontinue treatment		- As Grade 3, but consider starting Infliximab earlier

CTCAE: common terminology criteria for adverse events.

a HIV, hepatitis A and B, and blood quantiferon for tuberculosis, to prepare patients to start Infliximab.

#### Diagnosis and imaging findings

**Figure 3.**
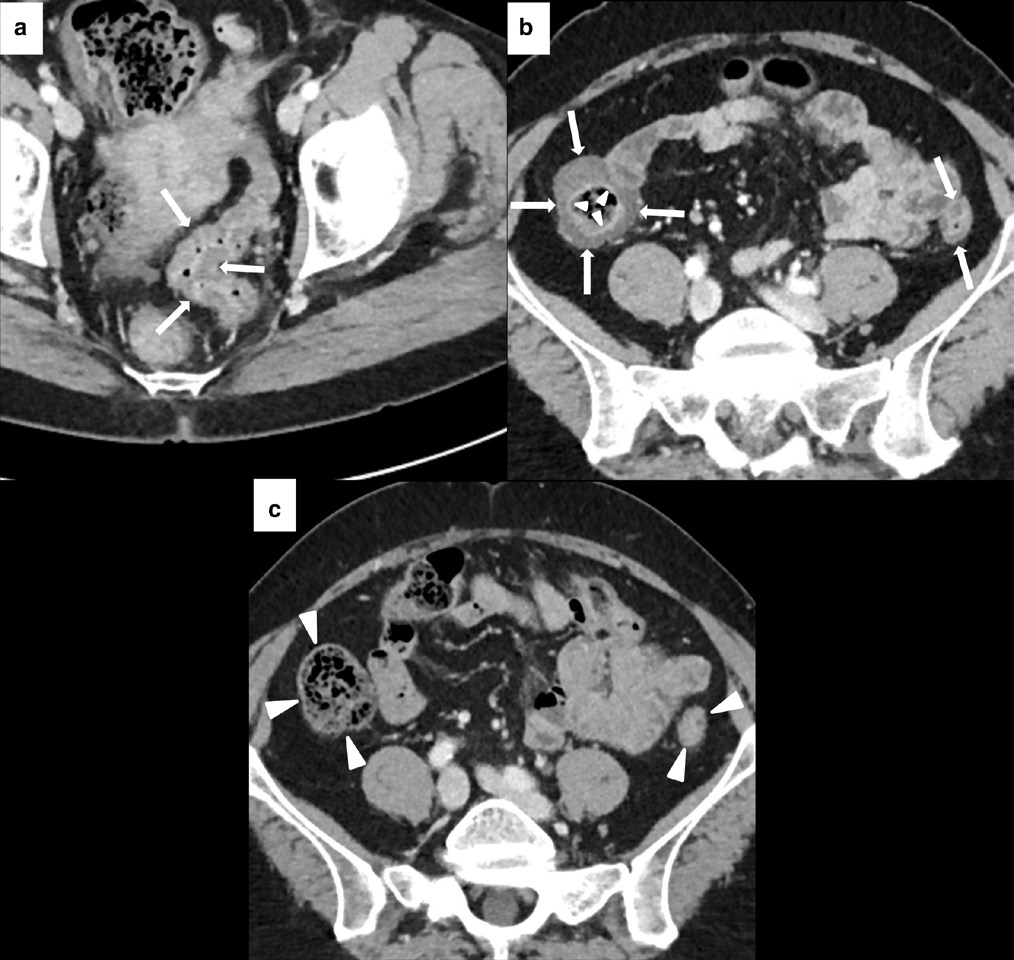
Imaging of diffuse immune-related colitis with a skipped distribution in a 49-year-old female with metastatic melanoma. After 4 months of treatment with Nivolumab (*n* = 12 cycles), patient presented with Grade 2 diarrhea and abdominal pain. CECT showed colonic wall thickening involving sigmoid (white arrows in A), descending and ascending colon (white arrows in B). Mucosal hyperenhancement was present in the ascending colon (white arrowheads in B). Nivolumab was discontinued until the resolution of the diarrhea and CECT after 4 months showed a normal colonic wall (white arrowheads in C).

**Figure 4.**
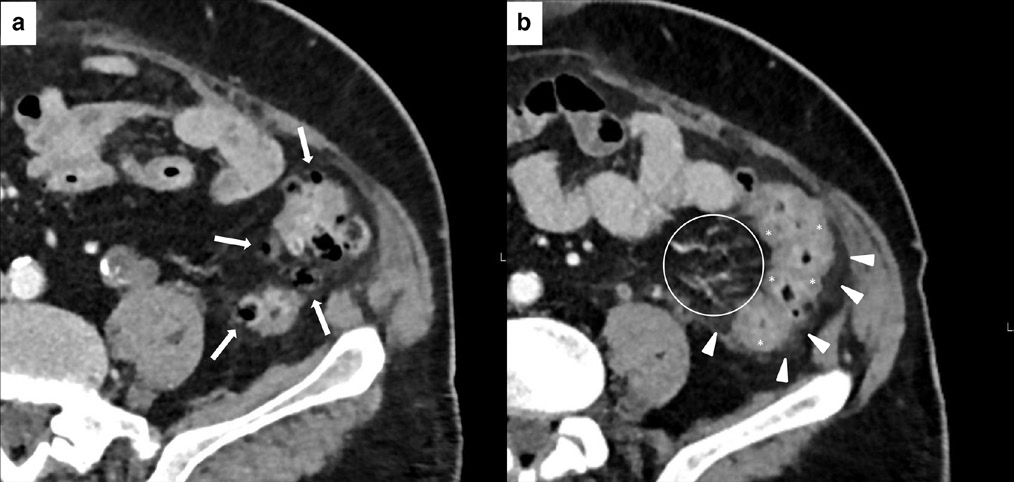
Imaging of SCAD of the descending-sigmoid colon in 76-year-old male with local and lymph nodal relapse of cutaneous angiosarcoma who presented lower abdominal pain and bloody diarrhea during nivolumab treatment. Axial CECT at baseline showed moderate wall thickening of the descending-sigmoid colon with underlying diverticulosis (white arrows in A). After 6 weeks of treatment, CECT showed a segmental wall thickening (white asterisks in B) with mesenteric vessel engorgement (area inside the white round in B) and pericolic fat stranding (white arrowheads in B).

**Figure 5.**
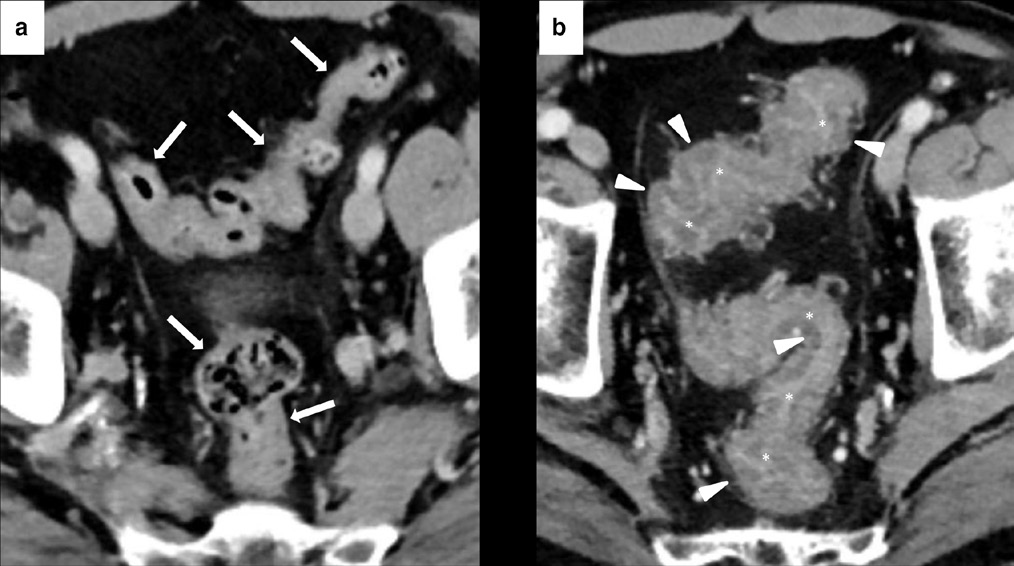
Imaging of isolated rectosigmoid colitis without diverticulosis in 59-year-old male with metastatic laryngeal carcinoma treated with pembrolizumab. Axial CECT at baseline did not show diverticulosis or abnormalities of the sigmoid and rectum wall (white arrows in A). After 4 months of treatment, patient presented lower abdominal pain and Grade 2 bloody diarrhea. CECT showed wall thickening of the sigmoid colon and rectum (white arrowheads in B) with mucosal hyperenhancement (white asterisks in B), without colonic fat stranding.

**Figure 6.**
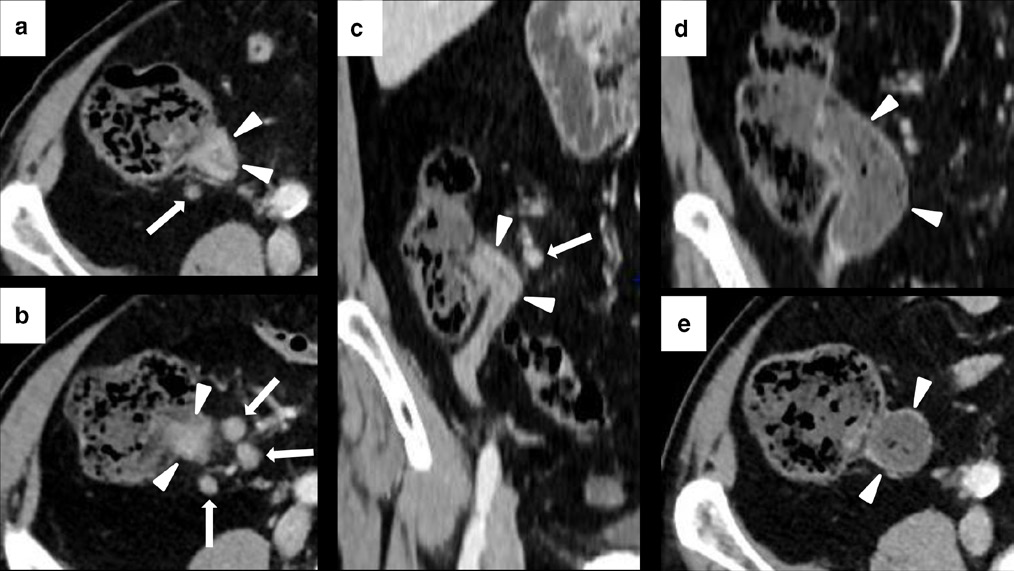
Imaging of terminal ileitis in a 80-year-old male with metastatic melanoma treated with nivolumab and history of inflammatory bowel disease (IBD). After 6 month of therapy, the patient presented abdominal distension and constipation. Axial CECT showed concentric wall thickening and mucosal hyperenhancement of the last ileal loop (white arrowheads in A, **B and C**) with some enlarged lymph node in the adjacent colonic fat (white arrows in A, **B and C**). Colonoscopy with biopsy was performed and histology showed a “terminal ileitis and intense chronic follicular inflammation associated with tiny ulcers, compatible with IBD”. Immunotherapy was not discontinued and the patient started a specific treatment for IBD. After 3 months, symptoms regressed and CECT showed a complete regression of the terminal ileitis (white arrows in D and E).

Colonoscopy with biopsy is the only diagnostic tools able to confirm immune-related colitis. Imaging, and in particular CT, is less invasive but also less accurate than endoscopy. Garcia-Neur et al^40^ correlated radiological finding with colonoscopy and colonic mucosa biopsy in patients with suspicions of Ipilimumab-induced colitis. CT was able to predict colitis in 96% of the 34 patients that performed both CT and biopsy. However, in the same series, the negative predictive value of CT was only 43% suggesting that imaging is not reliable in excluding colitis.^40^ In a second study by Tirumani et al, 22 of 28 patients (79%) treated with Ipilimumab and with a radiographically evident colitis performed colonoscopy and biopsy, which confirmed the ICI-related origin of the colitis.^41^ The following two main patterns of colitis were initially described with imaging: diffuse colitis and segmental colitis associated with diverticulosis (SCAD).^42^ Subsequently, Barina et al described a third pattern, the isolated rectosigmoid colitis without diverticulosis.^43^ Different imaging manifestations may be found (Table 2), more or less prevalent depending on the specific pattern.^36,37,42,43,45^ Diffuse colitis is characterized by colonic wall thickening that may have either a continuous or segmental distribution with skip lesions (Figure 3). Mesenteric vessel engorgement, mucosal hyperenhancement, and fluid-filled bowel distension are also common; profuse watery stool is predominant from the clinical point of view.^42,43^ CT findings in SCAD are limited to a segment of the colon (usually sigmoid), which usually contains pre-existing diverticulosis. It is also characterized by moderate wall thickening, mesenteric vessel engorgement (less frequent compared to diffuse colitis), and pericolic fat stranding (Figure 4). Clinically, mixed watery and bloody diarrhea and cramping pain are common. Relatively mild systemic symptoms and a negative stool test for bacterial pathogen or leukocytes may help to distinguish SCAD from active diverticulosis.^42,43^ In isolated rectosigmoid colitis without diverticulosis, the most common feature is the mucosal hyperenhancement with or without wall thickening, while the colonic fat stranding is not a characteristic of this pattern^37,43^ (Figure 5). Main differential diagnoses include Crohn’s Disease, ulcerative colitis, infectious, and pseudomembranous colitis^36^ (Figure 6). IRAEs of the upper GI tract present with thickening and edema of bowel wall at CT.^30,38,39^ However, diagnosis must rely on endoscopy followed by biopsy.^26,47^

**Table 2.** Main imaging manifestations of colitis

IMAGING MANIFESTATION	DESCRIPTION
Bowel wall thickening	Normal range of colonic wall thickness: from 1 to 2 mm when the lumen is well distended to 5 mm when the wall is contracted or the lumen is collapsed.^44^ Focal: extension <5 cm Segmental: extension of 6–40 cm Multisegmental: separate sites of segmental bowel wall thickening Diffuse: extension >40 cm.
Mesenteric vessel engorgement	Prominence or tortuosity of the vasa recta on CT (at the time of symptoms or compared to pre-treatment exam)^45^
Mucosal hyperenhancement	Increased enhancement of the colonic mucosa compared to other GI tract segments (*e.g.,* small bowel and stomach).^43^
Fluid-filled distended colon	Colonic dilatation: luminal diameter >8 cm for cecum and >6 cm for the rest of the colon.^45^
Pericolic fat stranding	Abnormal increased attenuation in pericolic fat, producing various appearances depending on the pathophysiologic process (ground-glass like, reticular pattern, reticulonodular appearance).^46^

PET imaging with 18F-FDG largely correlates with CT findings. A diffuse FDG uptake is usually present in diffuse colitis,^37,43,48,49^ while a segmental uptake may be present in SCAD and isolated rectosigmoiditis without diverticulosis.^37,43^ Since metformin may cause bowel mucosal FDG uptake, false-positives are a common pitfall in diabetics. Comparison with previous exams and correlation with medical history may help with the differential diagnosis. Discontinuation of metformin treatment 48 h prior the PET-CT scan should be considered if metformin-related bowel activity hampers the assessment of a suspected bowel pathology.^17^

### IRAEs - LIVER AND BILIARY TRACT

#### Incidence, clinical presentation, and management

Immuno-related hepatotoxicity (IRH) usually manifests itself between 3 and 14 weeks after the beginning of therapy with ICIs. Symptoms may begin earlier in patients treated with CTLA-4 antibodies.^44^ Their incidence depends on different factors, including ICIs class, dosage and whether mono- or combined treatment is performed. Incidence rate is lower with PD-1 antibody alone (0.7–2.1%), intermediate for PD-L1 antibody and/or standard dose of CTLA-4 antibody (0.9–12%) and higher using combined therapy (13%) or high dose of CTLA-4 antibody (16%).^44^ IRH may present with a wide spectrum of manifestations, from asymptomatic cases with a slight increase of liver function test without imaging abnormalities, occasionally associated with fever (most common clinical manifestation), to more severe cases with abdominal pain, appetite loss, weakness, fatigue, nausea, vomiting, and jaundice.^46,50–53^ Rare cases of fulminant hepatitis (0.1–0.2%) and liver-failure-related mortalities have been reported.^6,44,54,55^ Differential diagnosis includes idiopathic autoimmune hepatitis, viral hepatitis, and alcoholic liver disease.^36,51^ At histology, IRH may present with a prevailing hepatitis pattern (with hepatocellular injuries) or with a cholangitic pattern (with predominant bile ducts injury),^37,46,50,52,56,57^ the latter being less common (0.6%^58^). Immune-related cholangitis usually shows high level of cholestatic serum markers and a lower degree of transaminase level increase.^37,57,59^ Symptoms are similar to those of obstructive cholangitis, including general fatigue, abdominal discomfort or right upper quadrant pain, mild fever, nausea, and vomiting.^52,54,59,60^ Immune-related cholangitis shows a moderate-to-poor response to steroid therapy.^52,59–61^ According to the last version of CTCAE, severity of IRH is stratified in a five-grade scale (mortality related to hepatotoxicity is classified as Grade 5) according to hepatic markers levels, which increase proportionally with disease severity.^15^ Workup and management of the patients depend on the above-reported severity scale, as summarized in Table 3.

**Table 3.** Severity scale, management and workup of IRH(^19,20,23^)

	CTCAE Definition	Work-up	Management	Therapy
Grade 1	AST and ALT > 1–3 x ULN ALP and GGT > 1–2.5 x ULN Total bilirubine >1–1.5 x ULN	Weekly liver blood tests^a^	Continue ICI therapy	No treatment
Grade 2	AST and ALT > 3–5 x ULN ALP and GGT > 2.5–5 x ULN Total bilirubine >1.5–3 x ULN	Closer liver blood tests^a^ as severity increases Limit/discontinue hepatotoxic medications Liver screening tests and imaging to rule out other causes^b^ For Grade 3–4 consider hospitalization Only for Grade 4 consider liver biopsy	Drug-free period until liver blood tests recover to G1 levels	Consider corticosteroids if no improvement in 3–5 days and/or if adding symptoms
Grade 3	AST, ALT, ALP and GGT > 5–20 x ULN Total bilirubin >3–10 x ULN		Permanently discontinue treatment	Corticosteroids; if no response in 3 days consider adding Mycophenolate Mofetil
Grade 4	AST, ALT, ALP and GGT > 20 x ULN Total bilirubin >10 x ULN			

CTCAE: common terminology criteria for adverse events; AST: aspartate aminotransferase; ALT: alanine aminotransferase; ALP: alkaline phosphatase; GGT: gamma-glutamyltransferase; ULN: upper limit of normal.

a AST, ALT and bilirubin;

b Viral hepatitis, alcohol history, primary autoimmune hepatitis, iron studies, thromboembolic event, liver metastasis, primary malignancy.

#### Diagnosis and imaging findings

**Figure 7.**
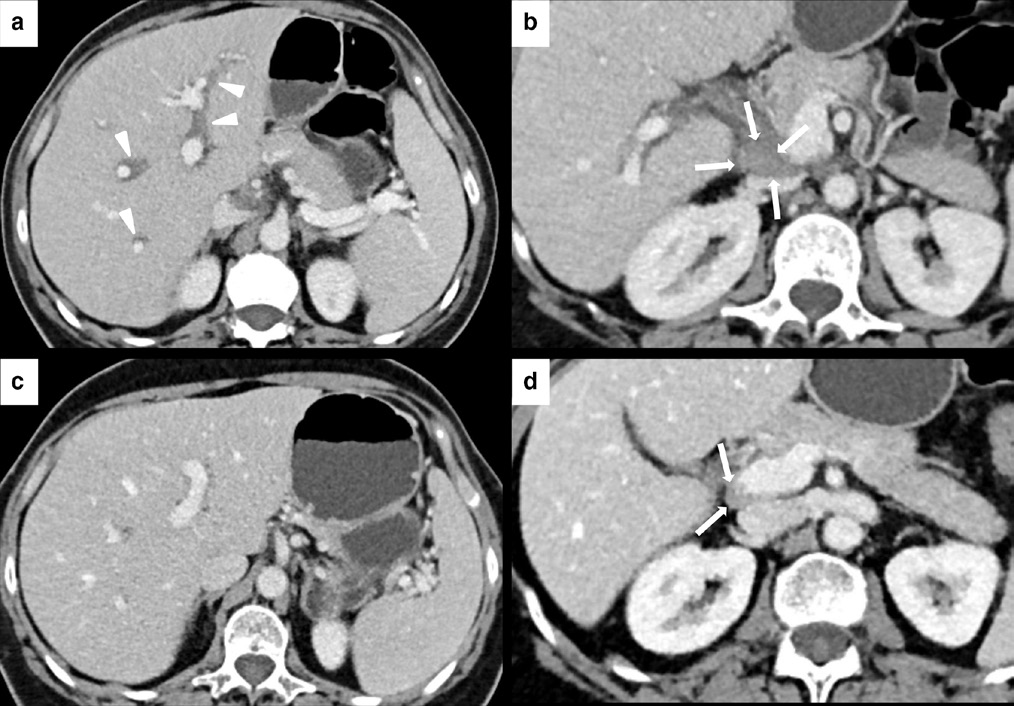
IRH in a 60-year-old female with renal cell carcinoma treated with nivolumab. After 3 months of therapy, the patient presented a slight elevation of gamma-glutamyltransferase (GGT) blood level and upper abdominal pain. Axial CECT showed periportal edema (white arrowheads in A) and new periportal enlarged lymph node (white arrows in B). CECT images after treatment discontinuation showed a complete resolution of the periportal edema (**C**) and the reduction of the periportal lymph node (white arrows in D).

As abovementioned, imaging is frequently uneventful in mild forms of IRH.^37^ Furthermore, differential diagnosis with acute hepatitis from other causes may be challenging, unless liver biopsy is performed.^37^ The most characteristic finding at US is the portal edema, which is characterized by a prominent echogenicity of portal vein walls or periportal spaces along with gallbladder wall edema and thickening.^36,37,46,48,54,62^ Hepatomegaly, steatosis, and lymphadenopathy have also been described.^48^ On CT, IRH typically presents with hepatomegaly, diffused parenchymal hypoattenuation or heterogeneous enhancement pattern with geographical areas of low-attenuation, which may obscure or mimic liver metastasis. Periportal edema and lymphadenopathy may also be present^36,37,45,46,50,54,62^ (Figure 7). Contrast-enhanced MRI may be helpful in distinguishing some IRH patterns from liver metastases. Periportal edema may present with an hyperintense signal of the portal vein wall and of surrounding tissues on *T*2-weighted(W) images.^36,37,54^

**Figure 8.**
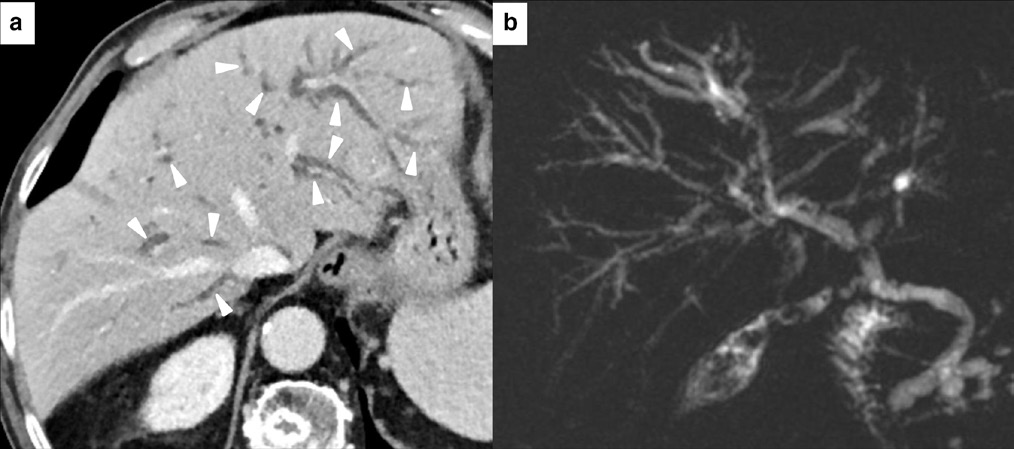
Imaging of immuno-mediated cholangitis in a 78-year-old male with metastatic melanoma, after 10 months of treatment with Atezolizumab. CT scan demonstrated bilateral intrahepatic bile duct dilatation (white arrowheads in A). Blood liver marker showed an increase of GGT, while transaminase levels were normal. MR cholangiopancreatography (**B**) confirmed CT findings and other causes of obstruction were not found. This pattern was classified as immuno-mediated cholangitis.

Bile duct injury may present as an extrahepatic predominant cholangitis showing different degrees of extrahepatic bile duct dilatation, usually without obstruction, and/or bile duct and gallbladder wall thickening and hyperenhancement. Otherwise, intrahepatic involvement may be predominant, showing dilatation or multifocal narrowing of the intrahepatic ducts^37,54,59^ (Figure 8). Detection of bile duct injury by imaging may forerun clinical presentation.^54,63^ Bile duct tree changes can be evaluated with endoscopic US or MR cholangiopancreatography.^37,54,59^ A last rare manifestations of bile duct involvement is the acalculosis cholecystitis, which is similar to typical acute cholecystitis from the clinical, management, and imaging points of view, with gallbladder distension, wall thickening, and pericholecystic inflammation without imaging detectable gallstones.^37,54,58,63,64^ Gallbladder wall radiotracer uptake may be seen on PET-CT.^54^ Liver biopsy may be requested for the differential diagnosis.^54,61^

### IRAEs – PANCREAS

#### Incidence, clinical presentation, and management

ICI-induced pancreatic injury (ICIPI) is an uncommon event that can occur up to 4% of cases during immunotherapy treatment.^65–67^ In a meta-analysis by George et al comprising 33 trials (16 with PD-1 inhibitors, 10 with CTLA-4 inhibitors, 4 with PDL-1 and 3 with the combination of PD-1 and CTLA-4 inhibitors) and 7702 patients, the incidence of all grade of lipase elevation was 4.17% for CTLA-4 inhibitors, 1.26% for PD-1 inhibitors, 14.29% for combination therapy, while the incidence of Grade 2 pancreatitis was 3.98% for CTLA-4 inhibitors, 0.94% for PD-1 inhibitors and 10.6% for combination therapy. No ICIPI-related mortality was reported.^66^ In a study performed by Abu-Sbeih et al among the 2.279 patients who had lipase level tested, the median time from ICI initiation and the peak of lipase elevation was about 9–10 weeks for CTLA-4 inhibitors, 15–16 weeks for combination therapy, and 20–21 weeks for PD-1/PD-L1 inhibitors.^67^ ICIPI may manifest itself with a wide spectrum of clinical events that can range from asymptomatic pancreatic enzymes elevation (amylase and lipase) to life-threating acute pancreatitis.^65^ Both pancreatitis and isolated increase of blood pancreatic enzymes are stratified in a scale grade of increasing severity (Grade 5 is the death of the patient) according to the latest versions of CTCAE^15^ or National Comprehensive Cancer Network (NCCN)^23^ guidelines (Table 4). Patient’s workup and management depend on these severity scales, as summarized in Table 4.

**Table 4.** Severity scale, management, and workup of ICIPI

		CTCAE/NCCN Guidelines Definition	Work-up	Management	Therapy
Pancreatic enzymes elevation	Grade 1	amylase/lipase > ULN−1.5xULN	Assess for signs/symptoms of pancreatitis	If no evidence of pancreatitis continue ICI therapy. Consider other causes.	No treatment If evidence of pancreatitis, manage according to pancreatitis algorithm.
	Mild Form	amylase/lipase ≥ 3xULN			
	Grade 2	amylase/lipase > 1.5-2xULN or >2.0–5.0 xULN and asymptomatic;	As Grade 1 and consider abdominal CECT or MRCP	As Grade 1 and evaluate if continue ICI therapy	
	Grade 3	amylase/lipase > 2.0–5.0 xULN with signs or symptoms or >5.0 x ULN and asymptomatic			
	Moderate Form	amylase/lipase > 3.0–5.0xULN			
	Grade 4	amylase/lipase > 5.0 xULN with signs or symptoms			
	Severe Form	amylase/lipase > 5.0xULN			
Pancreatitis	Grade 2	enzyme elevation or radiologic findings only	Assess for signs/symptoms of pancreatitis Provide basic medical support (hospitalization, aggressive fluid resuscitation, pain control)	As Grade 1 of “pancreatic enzymes elevation”. Evaluate gastroenterological counseling.	No treatment
	Mild Form	at least one among elevation of amylase/lipase > 3 xULN±radiological findings on CT ± clinical findings concerning for pancreatitis			
	Grade 3	severe pain, vomiting, medical intervention indicated		Hold ICI therapy	Prednisone/Methylprednisolone treatment
	Moderate Form	two or three among elevation of amylase/lipase > 3 xULN±radiological findings on CT ± clinical findings concerning for pancreatitis (*i.e.,* abdominal pain or vomiting)			
	Grade 4	life-threatening consequences, urgent intervention indicated		Permanently discontinue ICI therapy	Higher doses of prednisone/methylprednisolone treatment
	Severe Form	elevation of amylase/lipase > 3 xULN±radiological findings on CT ± severe clinical findings and hemodynamically unstable			

CTCAE: common terminology criteria for adverse events; NCCN: National Comprehensive Cancer Network; ULN: upper limit of normal.

Grade 1, Grade 2, Grade 3, and Grade 4: according to the last version (version 5) of CTCAE.^15^

Mild form, moderate form, and severe form: according to NCCN guidelines.^23^

Damage of the endocrine pancreas has also been reported, with an incidence of less than 1% of patients, most cases occurring in patients treated with PD-1/PD-L1 antibodies.^19,68–70^ Borroso-Sousa et al conducted a systematic review and meta-analysis on the incidence of endocrine dysfunction during ICI treatment, including 38 randomized clinical trials and a total of 7551 patients. Insulin-deficient diabetes was reported in only 13 cases, with six cases noted as Grade 3 or higher.^70^ Endocrine dysfunction of the pancreas after ICIPI may manifest itself with hyperglycemia, diabetes mellitus, or begin with ketoacidose.^23,69,71^ Also these events are classified in five grades or three forms (according to CTCAE^15^ and NCCN^23^ guidelines, respectively) mainly based on the glucose blood level.

#### Diagnosis and imaging findings

Imaging plays an important role in the diagnosis of ICIPI, in the assessment of its severity and in the identification of complications.^65^ Since amylase and lipase blood levels are not routinely performed, ICIPI may be occasionally identified at imaging in asymptomatic patients.^37^ Imaging findings of ICIPI are similar to those of traditional pancreatitis.^67^ It may present as either as an interstitial edematous pancreatitis (IEP) or a necrotizing pancreatitis (NP), and their related complications^65,72–74^ (Table 5). Of note, it might be difficult to distinguish IEP and NP at the initial examination. A new scan after 5–7 days might therefore be necessary for the differential diagnosis.^74^

**Table 5.** Summary of ICIPI patterns and its complications

	Interstitial Edematous Pancreatitis (IEP)	Necrotizing Pancreatitis (NP)
Early Complication (within 4 weeks)	Acute Peripancreatic Fluid Collections (APFCs)	Acute Necrosis Collections (ANCs)
Long-term Complications (after 4 weeks)	Pseudocyst	Pseudocyst Walled-off Necrosis (WON)

US has a limited role in the diagnosis of ICIPI since the evaluation of the pancreas is often hindered by patient’s habitus and the overlapping of bowel gas.^75^ However, US is often performed during the initial diagnostic workup to exclude the presence of biliary stones or biliary tree dilatation; in later disease phases, US may be useful to re-evaluate complications.^65^ Pancreatic enlargement and reduced echogenicity, due to focal or global parenchymal edema, represent the main US findings.^65^

**Figure 9.**
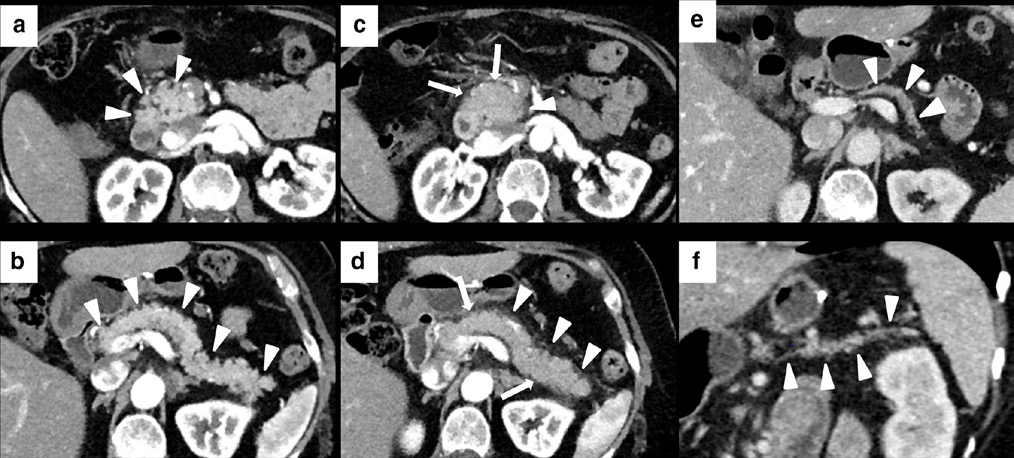
Imaging of ICIPI in a 61-year-old female with metastatic melanoma, treated with nivolumab. Baseline CECT showed normal appearance of the head (white arrowheads in A) and the body-tail (white arrowheads in B) of the pancreas. At follow-up after 4 months, patient presented dyspepsia, epigastric pain, diarrhea and elevation of lipase and amylase blood level. CT images showed a slight and homogeneous enlargement of the whole pancreas with loss of the physiologic lobulation (white arrows in C and D) associated with a subtle peripancreatic fat stranding (white arrowheads in C and D). This pattern was classified as IEP. ICI therapy was discontinued until normalization of blood lipase and amylase levels. Follow-up CT showed post-pancreatitis parenchymal atrophy (white arrowheads on axial and coronal CT, respectively, in E and F).

**Figure 10.**
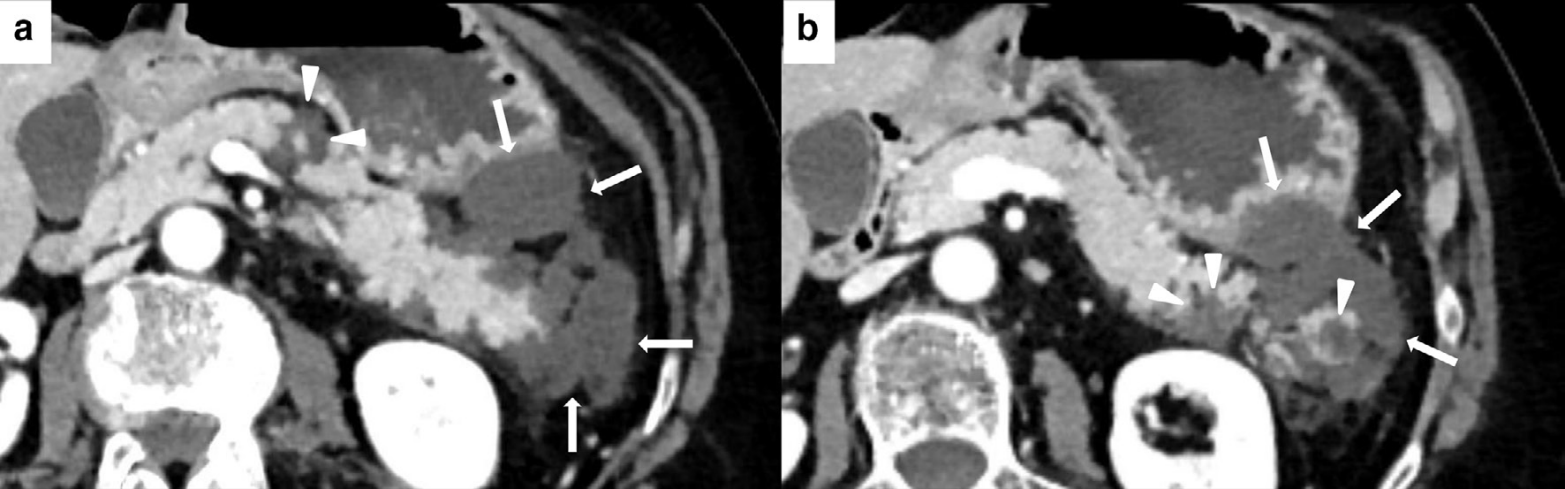
Imaging of NP in 62-year-old female during ICIs therapy for metastatic lung cancer. Axial CECT shows three necrotic unenhancing, slightly inhomogeneous, hypodense focal areas in the body and tail of the pancreas (white arrowheads in A and B), with necrosis of the peripancreatic fat planes (white arrows in A and B).

IEP represents the most frequent imaging finding of ICIPI. In this case, CT and MR may show focal, diffuse, or mass-like pancreatic enlargement, consequent to inflammation and edema, and either focal, diffuse, or heterogeneous decrease in the parenchymal enhancement. Peripancreatic inflammation may show as a stranding of the surrounding fatty layers^36,37,54,65,67,76^ (Figure 9). On MRI, edematous pancreatic regions are hypointense on pre-contrast T1W sequences, slightly hyperintense on T2W sequences and show restricted diffusion on DWI.^65,77,78^ Conversely, NP is characterized by the presence of necrotic areas involving the pancreatic gland or the peripancreatic spaces (alone or combined). NP appears as an inhomogeneous, well-defined non-enhancing area, hypodense on CT (Figure 10), hypointense on T1W images, and hyperintense on T2W images on MRI.^65,74,79^ Early complications (within four weeks) of IEP and NP are acute peripancreatic fluid collections (APFCs) and acute necrotic collections (ANCs). APFCs are localized in the peripancreatic space and they appear as homogeneous, due to the presence of fluid, hypodense on CT, hyperintense on T2W sequences and hypointense on T1W sequences on MRI, with no perceptible enhancement. ANCs are localized in the pancreas or in the peripancreatic space and they differ from APFCs for their heterogeneous content due to the presence of debris in addition to fluid.^65,79^ After four weeks, both APFCs and ANCs become encapsulated, with a uniform enhancing capsule containing only fluid (pseudocyst) or fluid, debris and loculations (walled-off necrosis).^65,67,74,77^ Necrotic areas may appear more inhomogeneous because of superinfection.^65^ Finally, ICIPI may result (or directly manifests^27^) as chronic pancreatitis, which can evolve in parenchymal atrophy^67,76^ (Figure 9).

On FDG-PET-CT, ICIPI is characterized by a focal or diffuse radiotracer uptake.^17,54,65,76^ Differential diagnosis with immunoglobulin G4-related autoimmune pancreatitis is a challenge. However, the latter presents usually in a focal form with loss of the normal fatty lobulations (described as “sausage pancreas”) and may involve other organs (biliary tract, salivary glands, aorta, and retroperitoneum).^36,76,80,81^

## Conclusion

Immune checkpoint blockades are becoming standard of care for an increasing number of cancer types. As consequence, an increased incidence of dysimmune toxicity, affecting different organs and systems, has been observed which will probably increase with time. IRAEs may impact on patient’s quality of life, may determine cancer treatment discontinuation and occasionally provoke life-threating or fatal events. For all of these reasons, early diagnosis and a prompt and multidisciplinary management of IRAEs is mandatory. GI tract, including liver and pancreas, is a very common site of IRAEs. Radiologists should be familiar with the timing of onset and the imaging patterns of GI IRAEs in order to promptly provide useful information to clinicians, to guide their treatment decision and to rule out other relevant diseases.
